# Novel Strategies to Optimize the Amplification of Single-Stranded DNA

**DOI:** 10.3389/fbioe.2020.00401

**Published:** 2020-05-05

**Authors:** Atef Nehdi, Nosaibah Samman, Vanessa Aguilar-Sánchez, Azer Farah, Emre Yurdusev, Mohamed Boudjelal, Jonathan Perreault

**Affiliations:** ^1^Medical Research Core Facility and Platforms, King Abdullah International Medical Research Center, Riyadh, Saudi Arabia; ^2^Department of Life Sciences, Faculty of Sciences of Gabes, University of Gabes, Gabes, Tunisia; ^3^Medical Research Core Facility and Platforms, King Saud bin Abdulaziz University for Health Sciences, Riyadh, Saudi Arabia; ^4^INRS-Centre Armand-Frappier Santé Biotechnologie, Laval, QC, Canada

**Keywords:** ssDNA amplification, asymmetric PCR, PCR by-products, inverted nucleotides, amplification of randomized DNA, nicking endonucleases

## Abstract

The generation of single stranded DNA plays a key role in *in vitro* selection of DNA aptamers and in other molecular techniques such as DNA sequencing and microarrays. Here we describe three novel methodologies for ssDNA production and amplification. Furthermore, we describe some previously unnoticed aspects of random DNA amplification. Our results showed that in asymmetric PCR the addition of a high melting temperature reverse primer blocked at its 3′ end by a dideoxy nucleotide drives the reaction further toward ssDNA production. We demonstrated also that incorporation of internally inverted nucleotide/(s) in one primer can be used as a new method of polymerization termination. Using such modified primer, the PCR product includes two complementary DNA strands having different lengths and separable from one another by denaturing gel electrophoresis. In addition, we showed that nicking enzymes can be used to cleave the undesirable strand allowing the isolation of the target ssDNA strand.

## Introduction

Aptamers are short single stranded oligonucleotides (DNA/RNA) that are inherently capable of folding into unique tertiary structures that selectively bind to a biological target with high affinity and specificity, making them potent tools for therapeutics, sensing, and synthetic biology (Rimmele, 2003; Marimuthu et al., 2012; Röthlisberger and Hollenstein, 2018). Aptamers are *in vitro* selected by a method referred to as Systematic Evolution of Ligands by EXponential enrichment (SELEX). The process comprises iterative rounds of library selection and amplification. It starts with the incubation of the target of interest with a large library of randomized single-stranded oligonucleotides, then elution of bound oligonucleotides followed by PCR amplification (Ellington and Szostak, 1990; Tuerk and Gold, 1990; Bock et al., 1992; Darmostuk et al., 2015). One critical step during the SELEX process of DNA aptamers is the generation of single-stranded DNA (ssDNA) from PCR products, since only ssDNA can form structural conformations to enable binding to the target molecules (Marimuthu et al., 2012).

Several methods have been described in the literature for this purpose including biotin-streptavidin separation, asymmetric PCR, size-based separation methods on denaturing polyacrylamide and enzymatic digestion with lambda exonuclease. The most common method used is separation of the desired DNA strand on streptavidin beads. One of the PCR primers is biotinylated at its 5′ end to generate the complementary DNA strand to the aptamer strand. Upon amplification completion the dsDNA product is immobilized on streptavidin beads. The desired non-biotinylated strand is eluted in denaturing alkaline conditions (Kilili et al., 2016). However, it was shown that this often results in dissociation of streptavidin causing multiple adverse effects (Paul et al., 2009). The eluate becomes contaminated with dsDNA (unable to bind to the target molecule) reducing enrichment efficiency. Moreover, the contaminant streptavidin present becomes another undesired target during the SELEX process and leads to subsequently enriching the selected pool with non-specific aptamers. To overcome these limitations, an alternative enzyme-based technique utilizing the 5′ to 3′ exonuclease selectivity of lambda exonuclease was developed. In this methodology, a 5′ phosphate group is introduced to the undesired complementary strand through a 5′ phosphorylated primer during PCR amplification (Kujau and Wölfl, 1997). Generated dsDNA is then incubated with lambda exonuclease which results in the degradation of 5′ phosphorylated strand and the release of single-stranded aptamer. Although this technique was reported to produce higher ssDNA yields, it requires optimization of enzyme concentration and incubation time to improve the quality of the ssDNA product (Avci-Adali et al., 2010).

Asymmetric PCR is designed to preferentially amplify one DNA strand. Thus it is useful when amplification of only one of the two complementary strands is needed such as in sequencing, hybridization probing and DNA-aptamer selection. The whole PCR process is similar to regular PCR, except that primers are added at unequal molar ratio to favor the synthesis of the desired DNA strand. This technique suffers from many limitations such as case-by-case optimization, low reaction efficiency and the frequent generation of non-specific product (Sanchez et al., 2004; Heiat et al., 2017). Furthermore limiting the concentration of one primer lowers its melting-temperature below the optimal reaction annealing-temperature which will complicate primer design.

In size-based separation methods, DNA strands with unequal size are produced as a result of chemical or structural modifications of one of the PCR primers (the primer complementary to the desired DNA strand) (Cao et al., 2009; Liang et al., 2015). The incorporation of a chemical spacer such as hexaethylene glycol (HEGL) (Williams and Bartel, 1995); constrained Nucleic Acids (CNA) (Martínez et al., 2011) or a GC-rich stem loop structure (Cao et al., 2009) at the 5′ end of the primer and downstream of poly-nucleotide extension, act as terminators of DNA polymerization. This leads to the production of a PCR amplicon partially double stranded, with two strands of unequal size that are separable on denaturing polyacrylamide gel.

In this study we describe three novel methods for the generation and amplification of ssDNA using constant (homogeneous) or randomized DNA template. We showed that the addition of a high melting temperature reverse primer blocked at the 3′ end by a dideoxy nucleotide increases the yield of asymmetric PCR. We also demonstrated that incorporation of inverted nucleotides in one of the PCR primers can be used as a new method of polymerization termination. A single internally inverted nucleotide incorporated through a 3′-3′/5′-5′ linkage downstream of an extra polynucleotide tail was sufficient to inhibit DNA polymerization. Hereby generating a dsDNA with two strands of different size which are easily separable by denaturing polyacrylamide electrophoresis (PAGE). Similarly, we demonstrated the use of one, or more, DNA nicking enzyme to allow purification of the chosen strand of DNA by denaturing PAGE.

## Materials and Methods

### Single-Stranded DNA Templates and Primers

All ssDNA oligonucleotides were purchased from Integrated DNA Technologies (IDT, United States). Two types of ssDNA oligonucleotides were used as PCR templates, either oligonucleotides with a fixed sequence or oligonucleotides with a central randomized region of 40 nt, flanked by two constant primer-binding regions. Supplementary Table S1 lists all primers and templates used with the corresponding sequences.

**R**everse **P**rimers with an **I**nverted **P**oly(**dA**) (**RPIPdA**) and **R**everse **P**rimers with different numbers of internally **I**nverted **dA**s (**RPIdAs**) were purchased from integrated DNA Technologies, Inc., United States. DNA oligos with internally inverted nucleotide(s) are not available on IDT catalog and they are offered as non-catalog option (on request). For these, annealing temperature had to be lowered from 72 to 65°C, as described in Section “Results.”

### Regular and Asymmetric PCR

Regular PCR reactions contained: 10 nM ssDNA template, 2 uM of Forward and Reverse primers, the volume was adjusted at 100 μl with MegaMix-Blue (μzone, United Kingdom). The thermo-cycling program used started with 1 cycle at 95°C for 5 min, followed by 30 cycles of 95°C for 30 s, 65°C for 30 s, and 72°C for 30 s.

Asymmetric PCR was performed in the same condition as regular PCR but with excess of Forward primer. The reverse (RP: limiting primer) and forward primer (FP: excess primer) were added at a ratio of 1:50 unless otherwise specified. DiDeoxy Reverse Primer (DDRP) modified asymmetric PCR was supplemented with DDRP at the same concentration as FP. DDRP was added at the end of cycle 10 (unless otherwise specified), if added at the beginning no PCR product was generated.

### Polyacrylamide Gel Electrophoresis (PAGE)

Products of PCR amplifications, with or without gel purification (Qiagen) were run on denaturing urea (8 M) polyacrylamide gel (8% w/v) in 1X TBE buffer following heat denaturation of samples at 95°C for 5 min and subsequent snap-cooling on ice. The electrophoresis was carried out at 25 W after pre-running the gel for 1 h. Typically, the gel was stained in 1X TBE containing 0.02% (v/v) SyberSafe (Invitrogen) and then imaged using ChemDoc (Bio-rad). Band intensity was analyzed using Image Lab software (Bio-Rad). When required, bands were eluted with a solution of 0.3M NaCl and ethanol precipitated.

### Native Agarose Gel Electrophoresis (NAGE)

Products of amplification were analyzed on 4% (unless otherwise stated) native agarose gel electrophoresis in 1X TAE buffer. SyberSafe dye (Invitrogen) was added to agarose at pre-casting step with a volume ratio of 1:10,000. DNA was detected using ChemDoc (Bio-rad) and band intensity was measured using Image Lab software (Bio-rad).

### 5′-Labeling of Primer Strands

100 pmol of DNA primers were 5′-^32^P-labeled using 10 U of T4 polynucleotide kinase (NEB) and 0.5 μl of [γ-32P] ATP (5 μCi), PNK buffer 1X (NEB) in a final volume of 10 μl. The mixture was incubated for 60 min at 37°C. The reaction was stopped by heating at 80°C for 10 min and purified by denaturing PAGE, the bands of nucleic acids were excised and eluted (see section “Polyacrylamide Gel Electrophoresis (PAGE)”).

### PCR Amplification of Nicking Assays dsDNA Template

For radioactive labeling, amplifications consisted of 50 μl reaction mixtures containing 2.5 mM MgCl_2_, 50 mM KCl, 10 mM Tris HCl (pH 8.3), 50 pmol of each primer (unlabeled), 200 μM each of the four dNTPs, 1.25 units of HotStart Plus Taq polymerase (Qiagen), 1 pmol of the DNA library and half of either the prepared labeled forward or reverse primers. Reaction mixtures were overlaid with 50 μl of mineral oil to prevent evaporation and contamination of the thermocycler. The amplification scheme was: 95°C denaturation for 7 min in the first cycle, 95°C denaturation for 30 s, 57°C annealing for 30 s and 72°C extension for 30 s for all subsequent 20 cycles, followed by a 72°C extension for 10 min in the last cycle.

For fluorescent labeling, amplifications of DNA consisted of 50 μl reaction mixtures containing 1.5 mM MgCl_2_, 50 mM KCl, 10 mM Tris HCl (pH 8.3), 0.5 μM of each primer (FP labeled in 5′ with Cy5, RP labeled in 5′ with fluorescein, from AlphaDNA, Inc.), 200 μM each of the four dNTPs, 1 units of Accustart II Taq DNA Polymerase (Quantabio) and 0.005 μM of the DNA library. The amplification scheme was: 94°C denaturation for 3 min in the first cycle, 94°C denaturation for 30 s, 58°C annealing for 30 s and 72°C extension for 30 s for all subsequent 25 cycles, followed by a 72°C extension for 3 min in the last cycle.

### Enzyme Digestion

Sequence of the library was designed to incorporate three nicking and two “standard” restriction enzyme cleavage sites (Supplementary Table S1). For single enzyme digestion, reaction was performed as suggested by manufacturer (NEB). For multiple enzyme digestion (*Kpn*I, Nb. *Bsm*AI, Nb. *Bts*I, Nb.BbvCi, and *Pst*I), 1 μl of DNA amplified fragments were digested by 10 U of each enzymes at 37°C for 1 h with 1X NEB Buffer 1.1 that contains (10 mM *Bis-Tris*-Propane-HCl, 10 mM MgCl_2_, 100 μg/ml BSA at pH 7). Then the digested fragments were heated to 80°C for 20 min for denaturation, and then chilled to 4°C. Note that all the chosen enzymes recognize a single sequence, ranging from five bases (Nt. *Bsm*AI: 5′-GTCTC-3′) to seven bases (Nb. *Bbv*CI: 5′-GCTGAGG-3′).

The digestion of fluorescent labeled DNA was heated, or not, 10 min at 94°C for denaturation and DNA complexes from all reactions were separated by native electrophoresis in a room at 37°C on a 3% agarose gel. Fluorescence was imaged with a Typhoon FLA9500. For denaturing conditions, urea PAGE was used as in Section “Polyacrylamide Gel Electrophoresis (PAGE).”

## Results and Discussion

### Amplification of ssDNA With Dideoxy Reverse Primer (DDRP) Asymmetric PCR

**FIGURE 1:**
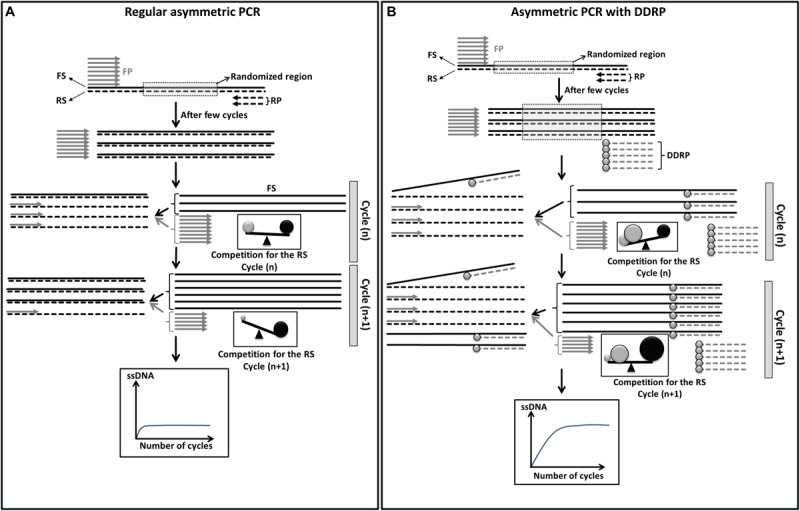
Schematic diagram showing theoretical improvement of asymmetric PCR yield by the use of a blocking primer. **(A)** Regular asymmetric PCR. The forward strands (FS, solid black lines) compete with the forward primer (FP, short gray lines) for the binding to the reverse strands (RS, dashed lines). In early cycles of amplification the competitiveness of FP (gray sphere) and FS (black sphere) are comparable due to the high concentration of FP and the length of FS. This will lead to equiprobable binding to the RS. With further cycles the quantity of FP decreases and the quantity of FS increases. This will lead to an increasing competiveness of FS however the competitiveness of FP will drop resulting in no more binding of FP to RS. In other words generation of FS will be blocked. Under such conditions the plateau of amplification of single stranded FS will be reached at very early cycles. **(B)** Asymmetric PCR with dideoxy reverse primer (DDRP). Addition of DDRP (short dashed gray lines with gray circle at the end) to the PCR reaction will limit the competitiveness of FS enhancing the binding of FP to RS which leads to the generation of higher quantity of single stranded FS. Under such condition the plateau of amplification of single stranded FS will be reached at later cycles.

In conventional asymmetric PCR (Figure 1A), after a few cycles of amplification, while the limiting primer (in this case the reverse primer) (Figure 1A: short dashed line) is being consumed, the newly produced complementary strand acts as a template for the excess primer [in this case the forward primer (FP)] to generate the desirable ssDNA strand [forward strand (FS)]. This full length higher melting temperature (Tm) FS competes with the shorter and lower Tm FP (in excess) for the limited reverse strand (RS) template. As PCR progresses, the quantity of generated FS gradually increases and leads to an increasing competition. Because of this uneven competition between the FP and the FS (Figure 1A), few cycles of asymmetric PCR will be enough to generate a small quantity of full length FS that will block completely the binding of the FP and subsequently block its own amplification.

To improve asymmetric PCR yield, the competition between the primer in excess and the generated ssDNA has to be adjusted to favor primer binding. We hypothesized that blocking of the nascent ssDNA with non-extendable complementary primer, like a DDRP, will limit its competition with the primer in excess for the complementary ssDNA template (Figure 1B). In such conditions, more PCR cycles and higher quantity of ssDNA has to be generated to reach the plateau of ssDNA amplification (amplification self-blocking).

**FIGURE 2:**
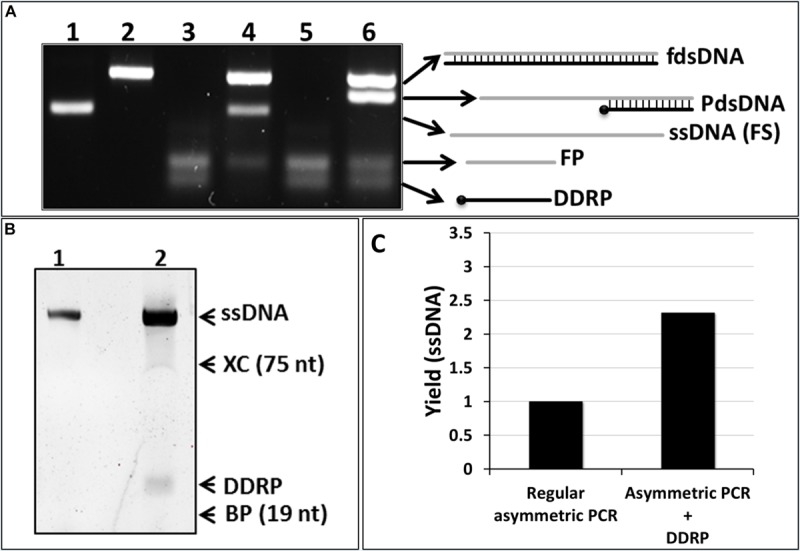
Conventional asymmetric PCR vs. asymmetric PCR with extra non-extendable primer (DDRP). **(A)** Native agarose gel (4% w/v) electrophoresis analysis (NAGE). (lane 1): single-stranded template (100 nt); (lane 2): regular PCR product; (lane 3): negative control reaction (primers only); (lane 4): regular asymmetric PCR at a ratio RP/FW of 1:10; (lane 5): asymmetric PCR with DDRP added at cycle 0; and (lane 6) at end of cycle 10 at FP/DDRP ratio of 1:1. **(B)** The ssDNA generated by regular asymmetric PCR (lane 4, **A**) and DDRP-asymmetric PCR (lane 6, **A**) was extracted, purified and analyzed by denaturing PAGE. **(C)** Quantification of generated ssDNA by regular asymmetric and DDRP-asymmetric PCR using ImageLab software.

In order to test this hypothesis, we performed two asymmetric PCR reactions. In these reactions FP was in excess (FP/RP = 10). One of the reactions was supplemented with the DDRP at a concentration similar to the FP. Analysis of the PCR products by NAGE showed two products in the normal asymmetric PCR, corresponding to the double and single stranded DNA products (Figure 2A, lane 4). However, in the reaction where DDRP was added neither double stranded nor fully single stranded DNA were generated (Figure 2A, lane 5).

We then realized that addition of the non-extendable DDRP at high concentration will out-compete the extendable reverse primer for the DNA template, hindering the synthesis of the reverse DNA strand and subsequently blocking further DNA polymerization (Figure 2A, lane 5). To overcome this problem, the reverse DNA strand, used as template for the FP in excess, has to be present in the reaction, either by using full dsDNA as template or by allowing its synthesis during the first few cycles of the PCR reaction. To do so, DDRP was supplemented to the asymmetric PCR reaction after the completion of 10 cycles. This resulted in fully dsDNA and a higher quantity of forward ssDNA product (Figure 2A, lane 6). The latter, however, migrated higher than the control ssDNA (lane 1); this is most likely due to the binding of DDRP (present at high concentration in the reaction) to the generated ssDNA leading to a partially double-stranded product. The ssDNA and partially dsDNA bands, resulting from asymmetric and DDRP modified asymmetric PCR respectively, were purified and analyzed on (8%) denaturing PAGE (Figure 2B). Results confirmed the identity of the amplified products shown as two bands of the same size of 100 nt corresponding to amplified ssDNA (Figure 2B, lanes 1, 2) and an additional band of 24 nt present only in DDRP-asymmetric PCR product (Figure 2B, lane 2) corresponding to the non-extendable primer DDRP. Results also confirmed our hypothesis and showed that addition of DDRP restrains the hybridization between the FS in excess and its complementary RS, leading to an increased availability of RS as template for the FP, thus leading to an increased yield. Under the conditions described above, addition of DDRP to asymmetric PCR reaction resulted in a yield-increase of ssDNA product higher than twofold (Figure 2C).

#### Improving DDRP Impact, on Asymmetric PCR Yield, by Increasing Its Blocking Efficiency

The higher the competition between FP and the FS for the reverse template, the lower is the amount of generated ssDNA. To improve asymmetric PCR yield the competitiveness of FP must be improved to the detriment of the nascent FS (Figure 1B). As we previously showed, the usage of a third non-extendable primer complementary to the nascent FS, during asymmetric PCR, limits its competitiveness favoring more binding of the FP and subsequently generation of more ssDNA.

**FIGURE 3:**
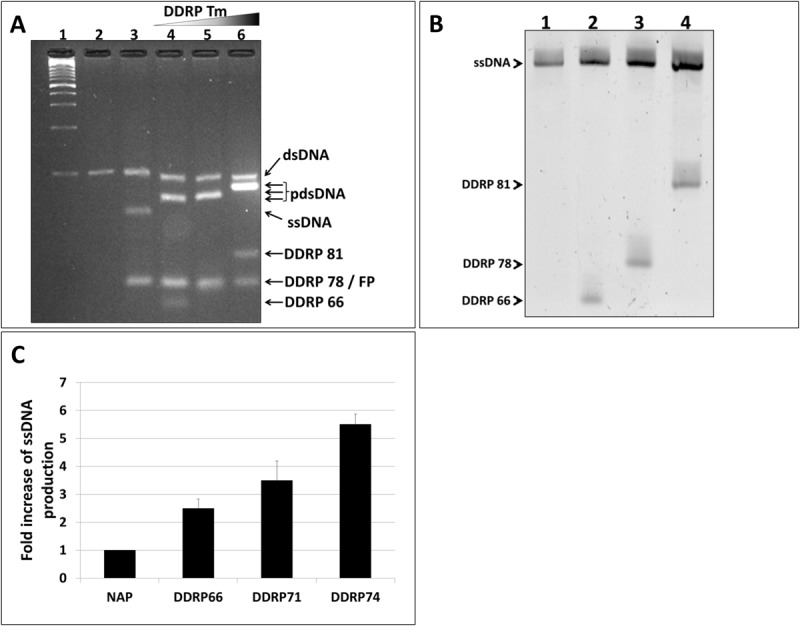
Effect of DDRP competitiveness (Tm) on ssDNA yield. **(A)** NAGE analysis (5% w/v) of DDRP-asymmetric PCR products performed with DDRPs having increasing Tm. (lane 1): 100 bp ladder, (lane 2): conventional PCR product, (lane 3): regular asymmetric PCR product (RP/FP ratio = 1:50) and (lanes 4–6): products of asymmetric PCRs performed with DDRP**_(Tm=66)_**, DDRP**_(Tm=78)_** and DDR**_(Tm=81)_** respectively. DDRPs were added at the end of cycle 10 (FP/DDRP ratio = 1:1). **(B)** All generated ssDNAs (lanes 3–6, **A**) were extracted from agarose-gel, purified and analyzed by denaturing PAGE (8% w/v). **(C)** Yield of ssDNA generated in asymmetric PCR reactions calculated from the polyacrylamide gel using ImageLab software.

It is axiomatic that the blocking efficiency of DDRP is proportional to its affinity to the nascent ssDNA. Thus, using DDRPs with higher melting temperature should improve ssDNA yield of asymmetric PCR. To prove this hypothesis, we designed and tested three DDRPs with different melting temperatures (66, 78, and 81°C). DDRPs were supplemented to PCR reactions after 10 cycles of amplification. RP/FP and FP/DDRP ratios used in these reactions were 1:50 and 1:1 respectively. It is to be noted that NAGE showed that all PCR reactions generated two populations of DNA: (i) 100 nt dsDNA corresponding to the higher band on the gel and (ii) a lower band corresponding to the ssDNA product hybridized to the DDRP primer. The partially ssDNA products displayed different migration patterns (shift) due to the varying lengths of the attached DDRPs (Figure 3A, lanes 4–6). Since the double stranded portion on these products have different lengths it is impossible to get a relative quantification based on ethidium bromide intensity. To accurately quantify the generated ssDNA by the different PCR reactions, partially dsDNA products were purified and analyzed by denaturing PAGE. Results confirmed that the partially dsDNA products are complexes of 100 nt ssDNA hybridized to the corresponding DDRP (Figure 3B). Gel-analysis showed also that the amount of generated ssDNA increases proportionally to oligo melting temperature, in other words DDRP blocking efficiency (Figure 3C).

**FIGURE 4:**
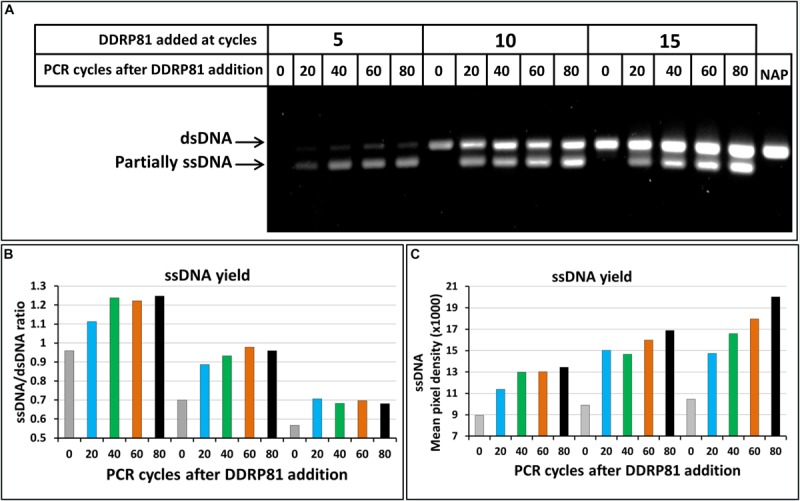
Effect of dsDNA template and PCR cycle number on ssDNA generation by DDRP-asymmetric PCR. **(A)** NAGE (5% w/v) analysis of products of asymmetric PCRs performed with DDRP_81_ added at the end of cycles 5, 10, or 15 and at 0, 20, 40, 60, and 80 cycles post DDRP_81_ addition. RP/FP and FP/DDRP_81_ ratios were 1:5 and 1:1 respectively. **(B)** Yields of different asymmetric PCR reactions in term of ssDNA/dsDNA ratio. **(C)** Yields of different asymmetric PCR reactions in term of absolute quantity of generated ssDNA. Band intensities were quantified using ImageLab software.

**FIGURE 5:**
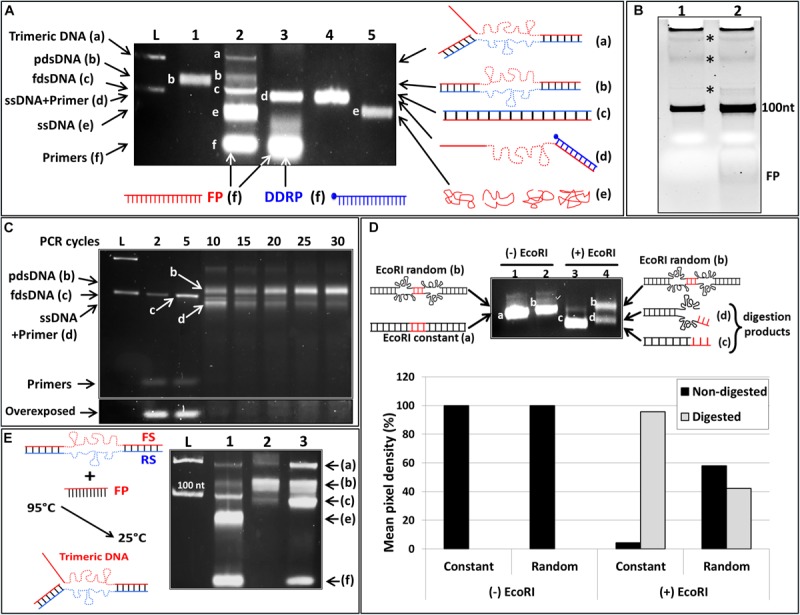
Effect of DDRP on the amplification of randomized ssDNA. **(A)** NAGE (5% w/v) analysis of asymmetric PCR product performed on a randomized ssDNA template with and without DDRP. (L): 100 pb ladder showing 100 and 200 bp bands, (lane 1): product of regular PCR performed on randomized DNA template (100 nt in length); (lane 2): regular asymmetric PCR reaction without DDRP showing five bands; band a: trimeric DNA, band b: pdsDNA, band c: fully double-stranded DNA (fdsDNA), band e: ssDNA and band f: primers. (lane 3): products of asymmetric PCR performed with randomized ssDNA template where DDRP was added at cycle 10. Product of this reaction resulted in two bands: band d (ssDNA hybridized to DDRP), and band f (free DDRP and FP); RP/FP and FP/DDRP ratios were 1:50 and 1:1 respectively. (lane 4): product generated by hybridization of randomized ssDNA with equimolar concentration of DDRP. Structures of different DNA moieties (bands) are schematically represented (on the right side). **(B)** Denaturing PAGE analysis of DNA products generated by regular PCR and asymmetric PCR performed on randomized DNA template. DNA products in lanes 1 and 2 of **(B)** are the same showed in lanes 1 and 2 of **(A)**. Stars indicate trace amount of by-products found in both regular and asymmetric PCRs. **(C)** NAGE (5%w/v) analysis of the structural evolution of asymmetric PCR product performed on randomized ssDNA. **(D)** Cleavage efficiency of two DNA templates containing a single *Eco*RI restriction site. One of these DNA is fully double-stranded, in the second the conserved *Eco*RI restriction site is flanked by two randomized sequences (schematic representation, on the left side). (lane 1): fully dsDNA; (lane 2): partially dsDNA; (lane 3): fdsDNA incubated with *Eco*RI; (lane 4): pdsDNA incubated with *Eco*RI. Cleavage products are schematically represented on the right side. Intensities of cleavage products by *Eco*RI were quantified by ImageLab software and represented as percentage of the starting material. Gray bars indicate the cleaved fraction and black bars indicate non-cleaved fractions. **(E)** Determination of band (a) structure. Experimental design is schematically represented on the left side. (lane 1): conventional asymmetric PCR performed with randomized ssDNA; (lane 2): regular PCR product performed with randomized ssDNA; (lane 3): product represented in lane 2 [band (b): pdsDNA] was hybridized with excess FP by heating at 95°C for 5 min followed by slow cooling to RT.

During the annealing steps of asymmetric PCR reaction, FP present in excess and the newly synthesized ssDNA (forward) compete for the binding to the RS template (Figure 1). A limited amount of the RS template will challenge and limit the chances of FP (weak competitor) to bind and subsequently this will limit the quantity of ssDNA generated by the asymmetric PCR. Based on this logic, increasing the amount of the RS template is expected to improve the yield in term of ssDNA. To increase the amount of RS template we can start with an increased quantity of dsDNA template but this option is not always possible especially in the case of DNA-aptamer selection where only ssDNA is available and has to be amplified. An alternative way to increase the RS template is to add the DDRP blocking primer later during PCR cycles. To assess the effect of the quantity of dsDNA template on DDRP-asymmetric PCR yield, we added DDRP81 blocking-primer at different amplification cycles (5, 10, or 15). Our results showed that the later DDRP is supplemented, the more ssDNA is generated (Figures 4A,C). Adding DDRP late during PCR cycles will lead to the improvement of the asymmetric PCR outcome in term of absolute quantity of ssDNA at the expense of ssDNA/dsDNA ratio (Figures 5B,C).

As opposed to classical PCR, in asymmetric PCR ssDNA amplification is not exponential but linear because only one primer is used. As a result, an increased number of amplification cycles should maximize ssDNA production. In classical PCR, due to the exponential amplification primers are usually consumed during the 20 first cycles of amplification, for relatively high template concentrations. However, in the case of asymmetric PCR supplemented with the blocking primer DDRP, the amount of generated ssDNA continuously increases over more than 80 cycles of amplification (Figures 4A–C).

#### Generation and Amplification of Randomized ssDNA Library by Asymmetric PCR

DNA-aptamers are identified through an iterative *in vitro* selection process, involving repeated selection/amplification cycles. The target DNA-aptamers are selected from a large random oligonucleotide library. ssDNA amplification plays a key role in this process. We showed in the previous section that addition of DDRP improved significantly the amplification of a constant ssDNA by asymmetric PCR. Here we investigated the effect of this strategy on the amplification of a randomized ssDNA template (Figure 5). Beside asymmetric PCR with DDRP (Figure 5A, lane 3) we performed a classical PCR (Figure 5A, lane1) and conventional asymmetric PCR (Figure 5A, lane 2) reactions as controls for size and yield respectively. RP/FP ratios of classical and asymmetric PCR were 1:1 and 1:50 respectively. NAGE showed that the product of classical PCR migrated higher than the expected size (100 bp) and the band appeared diffuse. It is expected that during early cycles of PCR, where primers are found in enough quantity, ssDNA template would be perfectly filled leading to a fully double stranded DNA product. However during late PCR cycles, where primers are completely consumed, no polymerization occurs, dsDNA product will simply be subjected to repetitive cycles of denaturation/annealing. This could result in a random hybridization of reverse and forward DNA strands leading to a partially dsDNA product (pdsDNA). pdsDNA will be perfectly double stranded at the constant regions in the 3′ and 5′ ends but the central randomized region will be partially double stranded with many bulge-motifs in non-complementary segments. This will result in the formation of different DNA structures and thereby different migration profiles during NAGE.

To verify this hypothesis, a comparative analysis of PCR products over late and early cycles was performed (Figure 5C). Fractions of PCR product were collected at different cycles just after the elongation step. Analysis with NAGE showed that these products of early cycles (cycles 2–5) were perfectly double stranded as indicated by the migration pattern (sharp thin band migrating at exactly 100 pb) [Figure 5C, cycles 2, and 5 band (c)]. At cycle 10, the PCR primers were almost completely consumed (Figure 5C, overexposed part), as such no further amplification occurred, and rather the DNA product underwent iterative denaturation/annealing cycles. Since the DNA is randomized in its central region, the probability that the FS would find its complementary RS is very low. The flanking constant regions at the 3′ and 5′ ends drive a random annealing of DNA strands leading to the formation of pdsDNA shown on the gel as a shifted diffuse band [Figure 5B lanes 10–30 top band (b)]. To further support this, we inserted a single *Eco*RI restriction site in the randomized region of the random DNA template (*Eco*RI random oligo); we used a constant DNA template of the same size and with a single *Eco*RI restriction site at the same position (*Eco*RI constant oligo) as a positive control. We performed a classical PCR then DNA products were purified and subjected to *Eco*RI digestion. Cleavage of PCR products by *Eco*RI constitutes evidence that these products are perfectly double stranded. Analysis of PCR products, digested with *Eco*RI, by NAGE showed that the control dsDNA product is fully digested (Figure 5D, lane 3) however the randomized product was partially digested (40%) (Figure 5D, lane 4). This indicates clearly that the randomized central region is not perfectly double stranded. This might be due to the fact that the *Eco*RI restriction site within the randomized region is not accessible to the restriction enzyme because of the flanking DNA motifs or it is not formed because of its low Tm especially that we performed the digestion reaction at 39°C.

Using the randomized DNA as template, asymmetric PCR produced five bands (a–f) (Figure 5A, lane 2). It is reported in the literature that amplification of random DNA libraries with conventional asymmetric PCR generates a multitude of products (bands) known as by-products (Sanchez et al., 2004; Venkatesan et al., 2013; Tolnai et al., 2019). It is suggested that during amplification, non-specific hybridization to the random region serves as a primer for DNA polymerization and subsequently yielding a buildup of longer DNA products (Musheev and Krylov, 2006). This mechanism may lead to longer DNA by-products, even before the primers are exhausted (Tolle et al., 2014). We showed previously, that conventional PCR performed on random DNA template generates a pdsDNA with shifted migration on NAGE not due to size but due to structure. Thus we believe that the so-called by-products generated by asymmetric PCR on random DNA template, are constituted of DNA moieties of the same size but displaying different mobility patterns because of different structural arrangements. The five bands generated by the asymmetric PCR (Figure 5A, lane 2) include band (e), which corresponds to ssDNA as it has a similar mobility as the control ssDNA (Figure 5A, lane 5) as well as band (a), which showed the lowest migration. We expected that, due to the high concentration of FP, a trimeric DNA could be formed by hybridization of this primer to the pdsDNA (b) (as illustrated in Figure 5E, schematic representation). To support this hypothesis, and to allow the formation of the trimeric DNA, we mixed the pdsDNA, generated by traditional PCR on random DNA template (Figure 5A, lane 1), with an excess of FP. The mixture was heated at 95°C for 5 min then slowly cooled down at room temperature to allow annealing. On NAGE, the mixture resulted in four bands (Figure 5E, lane 3): Band (f) corresponds to the FP in excess, Band (c) migrated at the good size of 100nt and corresponds to the perfectly double stranded DNA, Band (b) correspond to the pdsDNA and a fourth band migrating together with product (a) of the conventional asymmetric PCR. This result indicates that the product (a) is not a result of amplification but a result of hybridization of the pdsDNA and the FP. Since the pdsDNA is not perfectly annealed the FP found at high concentration competes easily for its binding-site leading to a DNA complex formed by the FS, the RS and the FP (trimeric DNA).

It is worthy of note that the heated mixture (Figure 5E, lane 3) generated not only a trimeric DNA (band a) but also a high amount of perfectly dsDNA (band C). This might be caused by two factors. The first might be the slow cooling which allowed a perfect hybridization of complementary strands. The second might be the high competitiveness of the FP due to its high concentration, which once attached to its binding site on the RS, can only be displaced by a perfectly complementary FS. This will lead to the formation of a fully dsDNA.

It is worthy of note also that asymmetric PCR performed with random DNA template (Figure 5A, lane 2) generates more ssDNA than asymmetric PCR performed with constant DNA template (Figure 3A, lane 4). A higher quantity of generated ssDNA is directly proportional to the competitiveness of the FP (in excess). Since the same FP was used in these two reactions at the same concentration, the higher quantity of generated ssDNA by asymmetric PCR on random template testifies that FP binds more easily to the RS template when a random DNA template is used. This difference between the ease with which random libraries can be used to produce ssDNA compared to fixed sequences also support the fact that the product of asymmetric PCR on random template is not perfectly double stranded which allows more binding of FP and subsequently the generation of higher quantity of ssDNA.

Asymmetric PCR is often used for the amplification of random ssDNA during DNA-aptamer selection. We wanted to evaluate whether the differences between amplification of constant and random sequences would impact the use of a non-extendable reverse primer (DDRP) during asymmetric PCR for ssDNA production. Briefly, a randomized DNA template was used and the DDRP was added to the PCR reaction after 5 cycles of amplification. PCR product was analyzed with NAGE and resulted in only two bands (d) and (f) (Figure 5A, lane 3). The Band (f) corresponds to primers in excess (FP and DDRP), the Band (d) was not observed among products of asymmetric PCR without DDRP (Figure 5A, lane 2) and migrates faster than the perfectly dsDNA (band c) but slower than the ssDNA (band e). Since this product (band d) is linked to the presence of DDRP during asymmetric PCR, we expected that it is a DNA complex formed by the hybridization of DDRP (in excess) to newly generated ssDNA (FS) as previously observed in Figures 2A, 3A. The composition of this product (band d) was confirmed by annealing equimolar ratios of single stranded forward DNA and DDRP primer. The mixture was heated at 95°C for 5 min to ensure full denaturation then slowly cooled down to room temperature to allow hybridization. The product of this hybridization (Figure 5A, lane 4) migrated exactly at the same level as the DDRP-asymmetric PCR product, confirming our hypothesis.

It is worthy of note that addition of DDRP to asymmetric PCR performed on random DNA template generated only one product (band d), all the other “by-products” (a, b, and c) were not observed. We showed previously that all of these “by-products” have different migration patterns not because of their sizes but because of their structures. Addition of DDRP seems to enable the formation of all of these alternative structures in the favor of the structure (d). We already showed that the FP binds more efficiently to its template (RS) when a random DNA is used as template (the random FS is less competitive), moreover the addition of DDRP that binds to the forward DNA strand will limit further the competition of this latter with the FP for binding to the random RS. In such conditions, higher quantity of single stranded forward DNA will be produced. On the other hand, the melting temperature (Tm) of the DDRP is higher than the Tm of the regular reverse primer (RP), and this will lead to complete blocking of RS synthesis when DDRP is added to PCR reaction (after few cycles). Since the RS is needed for the formation of all “by-products” (a, b, and c), its low concentration will not enable the formation of these structures, but instead will favor structure (d) formed by hybridization of DDRP and the forward DNA strand (both found in excess).

In conclusion, addition of a non-extendable primer to asymmetric PCR improves not only the quantity of generated random ssDNA but also its quality through the inhibition of alternative by-products formation.

### Generation and Amplification of ssDNA Using Inverted Nucleotides as Terminator of DNA Polymerization

To generate and amplify ssDNA, another strategy was developed. This strategy is based on the amplification of the dsDNA by PCR and then the separation of the suitable strand from the amplified product. This strategy will be impossible in the case of conventional PCR because the reverse and forward strands have exactly the same molecular weight (MW). To make their separation possible by denaturing gel electrophoresis, the two strands of the amplification product have to be of different MWs. To generate such PCR product, modified PCR primers with an extra polynucleotide tail attached through a DNA-polymerization stopper were used. During DNA amplification, the polymerization stopper downstream of the polynucleotide sequence will not be recognized by the DNA polymerase and induces its early detachment, preventing synthesis of the complementary sequence of the extra polynucleotide tail. Under such condition, only one of the DNA strands of the amplification product will contain the polynucleotide tail and subsequently will have a higher MW. Then the two DNA strands could be easily separated using size-based DNA purification. A commonly used DNA-polymerization stopper is *Hexaethylene glycol* (HEGL). HEGL is an 18 atom spacer that can be placed at 5′, 3′ or internally in a DNA sequence. This spacer is not compatible with DNA polymerization and it induces DNA polymerase detachment and subsequently it acts as a polymerization stopper. Despite its efficiency and stability HEGL remains a non-DNA moiety. In this part of this study we successfully used only nucleotides to stop DNA polymerization and subsequently generating PCR products where the two strands have different MWs.

**FIGURE 6:**
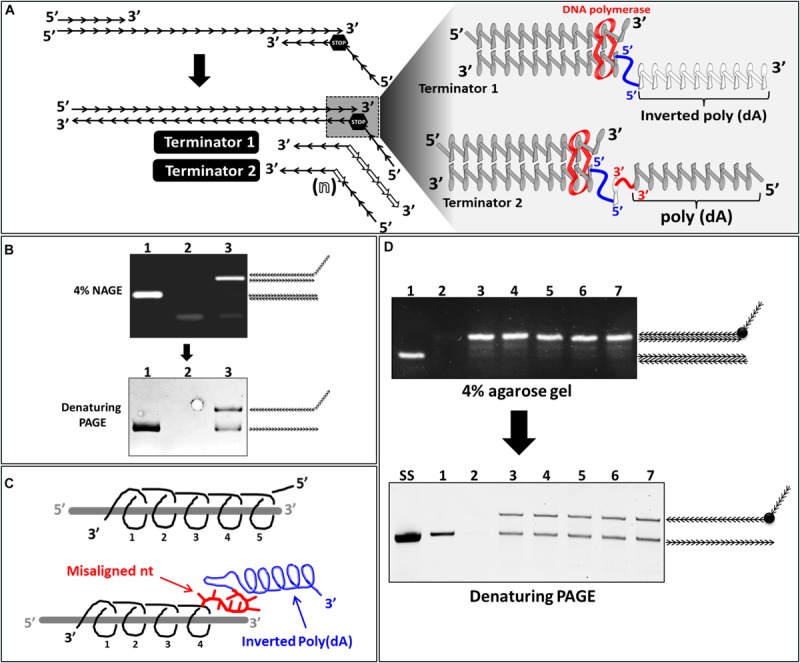
Effect of incorporation of inverted nucleotide/s on ssDNA generation. **(A)** Schematic illustration of the reverse primer with either an inverted poly(A) tail (terminator 1) or internally inverted nucleotide/s (terminator 2) and the predicted effect of these moieties on DNA backbone and DNA polymerization (magnified). **(B)** (lane 1): regular PCR product performed on a constant DNA template (100 nt in length); (lanes 2 and 3): products of PCR performed with a reverse primer containing an inverted poly (dA) (Terminator 1) at annealing temperature of 72 and 60°C respectively were analyzed with NAGE (top) and PAGE (bottom). **(C)** Schematic illustration of the effect of the inverted poly(dA) tail (gray helix) on RP (black helix) backbone structure. **(D)** NAGE (4% w/v) (left side) and PAGE (8% w/v) (right side) analysis of PCR products performed with reverse primers containing different numbers (1 to 5) internally inverted dAs. (lane 1): product of regular PCR; (lane 2): negative control (no template); (lanes 3 to 7) Products of PCRs performed with RP containing 1, 2, 3, 4, or 5 internally inverted dA/s downstream of a poly(dA) tail (non-inverted) (Terminator 2).

It is well-established that DNA polymerase requires a free 3′OH group for synthesis initiation. DNA synthesis can be made in only one direction by extending the 3′ end of a pre-existing primer moving on the template strand from its 3′ toward its 5′ end. We exploited this property of DNA polymerase to investigate whether a completely inverted polynucleotide (Figure 6A, terminator 1) or incorporation of internally inverted nucleotide/s (Figure 6A, terminator 2) to which a downstream polynucleotide tail connected through a 3′-3′ linkage could be used as a new strategy to stop DNA polymerization during PCR.

We therefore assessed the efficiency of **R**everse **P**rimer with an **I**nverted **P**oly(**dA**) (**RPIPdA**) in stopping DNA polymerization during PCR. A standard RP without poly(dA) tail was used as a control. The initial PCR reaction performed with an annealing temperature of 72°C generated in the control reaction a dsDNA product with the expected size (100 nt) (Figure 6B, lane 1), however in the reaction where an inverted poly(dA) tail was added to the (RP) no product was detected (Figure 6B, lane 2). This suggests that the additional inverted poly(dA) inhibited somehow the annealing of the RPIPdA. A possible explanation might be that the inverted poly(dA), which has an opposite helicity compared to the RP to which it is linked, might induce a distortion of the DNA backbone of this latter and subsequently a misalignment of neighboring nucleotides at the 5′ end of the RP (Figure 6C). Misaligned nucleotides of the RP will not be able to anneal to the DNA template and subsequently the real Tm of the (RP) will be lower than the theoretical. To overcome this problem we performed a new PCR reaction with a reduced annealing temperature (60°C instead of 72°C), results showed that under these conditions the DNA template was perfectly amplified leading to a dsDNA product with the expected size (100 nt) (Figure 6B, lane 3). Denaturing PAGE Analysis of PCR product performed with RPIPdA resulted in the generation of two bands with different MWs (Figure 6B, lane 3). These results confirmed that the inverted poly(dA) disturbed the binding of RP but more importantly it induces DNA-polymerization termination.

We then tested the minimal number of inverted nucleotides required for stopping DNA polymerization. We designed different **R**everse **P**rimers with different numbers of internally **I**nverted **dA**s (**RPIdAs**) downstream of a poly(dA) tail (not inverted in this cases) (Figure 6A, Terminator 2). PCR products resulting from the use of these primers, along with the product of the control PCR performed with standard FP and RP, were analyzed by NAGE and denaturing PAGE. Results showed that, in NAGE, all PCR reactions performed with RPIdAs generated dsDNA products (Figure 6D, lanes 3 to 7) higher than the control dsDNA (100 nt) (Figure 6D, lane 1) due to the incorporation of the additional poly(dA) tail. In denaturing PAGE the control dsDNA resulted in only one band corresponding to the forward and reverse strands of the same length. However all dsDNA products of PCR performed with RPIdAs resulted in the generation of two bands each indicating that the forward and the RS in these products are of different length. This result indicates clearly that only one internally inverted nucleotide inserted in RPIdA downstream of the poly(dA) tail is enough for stopping efficiently DNA polymerization.

In conclusion, we showed in this part of the study that an inverted poly(dA) tail or an internally inverted nucleotide stop efficiently DNA polymerization during PCR leading to a dsDNA product where the forward and reverse strand are of different length. This methodology could be used for ssDNA amplification and may replace methodologies where non-DNA polymerization stoppers are used.

### Generation of ssDNA Using Nicking Enzymes

**FIGURE 7:**
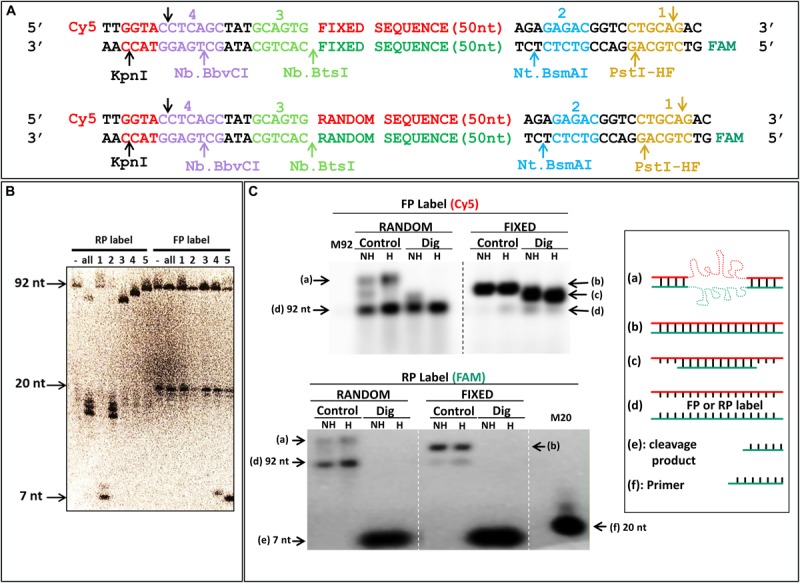
Specific digestion of reverse strand DNA. **(A)** Scheme of the designed library, and control “fixed” sequence, with cleavage sites of each enzyme pictured in a different color in the constant or random sequence regions with fluorescent labels; “Cy5”: cyanine (Forward); “FAM”: fluorescein (Reverse). **(B)** Denaturing urea PAGE of digested DNA. Numbered sites in **(A)** correspond to the enzymes used in the lanes of the gel; RP label and FP label: radioactive labeling of reverse strand via the labeled reverse and forward primers, respectively; “-”: uncleaved PCR; “all”: all three nicking enzymes (2, 3, and 4) together with *Kpn*I or *Pst*I-HF, in RP or FP label, respectively. Full length DNA is 92 bases; the 20 bases band correspond to labeled primers; and 7 bases band to either *Kpn*I (FP) or *Pst*I-HF (RP) cleavage products. **(C)** Native agarose gel of DNA with fixed and random sequences. Each PCR amplicon was labeled with fluorescence as indicated in **(A)** and imaged with a Typhoon FLA9500 for Cy5 (FP label) and fluorescein (RP label). M92: marker ssDNA, 92 bases; “M20” Marker ssDNA, 20 bases correspond to labeled primer; “Control”: uncleaved PCR; “Dig”: digestion with all three nicking enzymes (2, 3, and 4) together with *Pst*I-HF, in reverse primer label; full length DNA is 92 bases; and 7 bases band corresponds to *Pst*I-HF (RP) cleavage products; NH: samples were not heated before loading; H: samples were heated to 94°C before loading; “a–f”: different structures of DNA and markers used are schematically represented (on the right side, as in Figure 5). Electrophoresis was done in a room at 37°C in a 3% NAGE (native agarose gel).

In parallel, we were interested in finding a way to amplify and generate FS ssDNA with standard primers. The commonly used HEGL primers, as well as the primers including inverted nucleotides, require unnatural nucleotide modifications. On the other hand, if it was possible to use enzymes that specifically degrade only one strand, it would be possible to use it for ssDNA generation. Lambda exonuclease has been used for such purpose, but this also requires a modified oligonucleotide because the selectivity of the enzyme is based on presence of a 5′-phosphate, and this selectivity is not perfect either. Most restriction enzymes are very specific for their cognate site, but because they cleave both strands, they would provide only a small size change as compared to the complementary strand. Conversely, nicking enzymes cleave only one strand, while still being very specific for their cognate site. We thus included nicking restriction sites in the constant sequences of the primer binding sites of our library; for our templates we used two long oligonucleotides of 92 nt (see also Supplementary Table S1), both a constant sequence (Figure 7A, top) and an oligonucleotide with a randomized middle region (Figure 7A, bottom). All three nicking enzymes cleaved the RS (Figure 7B, RP label, lanes 2–4), while leaving the FS intact (Figure 7B, FP label, lanes 2–4). Even if the gel pictured in Figure 7B is a 20% denaturing PAGE, separation of FS from RS is relatively easy (and would be even easier on a lower% gel). As noted by the smaller digestion products when enzymes are combined (Figure 7B, RP label, lane “all”), the size difference is even larger in this case. Additional “standard” restriction enzymes (*Pst*I-HF and *Kpn*I) were included as well to provide further fragmentation potential of the constant DNA sequence, as well allow ligation and cloning if useful or necessary. Note that even if in principle using a single nicking enzyme suffice to distinguish between the forward and reverse strands, as shown by differences in migration for bands with RP label compared to FP label (Figure 7B), using two enzymes, such as 2 and 3 (Figure 7B) will provide a larger size difference (92 vs. 50 + 22 + 20 bases) which will make it even easier to distinguish on gel. Furthermore, the native gel in Figure 7C, also corroborates the differences between fixed sequences and random libraries, even suggesting that denaturing gel purification might not be absolutely required for strand separation, as indicated by the unique band corresponding to FS ssDNA when digested sample is heated (Figure 7C, random, dig, “H”). Interestingly, even in absence of digestion, a significant proportion of FS from the random library dissociates to become single strand when run at 37°C (Figure 7C, Random FP and RP labels).

## Conclusion

Several methods exist to produce ssDNA from PCR products (Williams and Bartel, 1995; Kujau and Wölfl, 1997; Cao et al., 2009; Avci-Adali et al., 2010; Martínez et al., 2011; Liang et al., 2015; Kilili et al., 2016), but each method as some associated limitations (Sanchez et al., 2004; Paul et al., 2009; Heiat et al., 2017). We therefore provide three additional approaches for the production of ssDNA, which may have some limitations as well, but also have advantages over some of the existing methods. The improvement of asymmetric PCR with DDRP significantly increases yield. Although minor, the main limitation compared to typical asymmetric PCR is that the DDRP should be added after 10 initial cycles of PCR. As for the RPIdAs, they do not require the addition of chemical groups not found in standard nucleic acids compared to the HEGL primers more often used for ssDNA preparation from denaturing PAGE. Ironically, one of the limitations is that these are not standard modifications and thus not offered by all oligonucleotide suppliers. A way to circumvent the use of non-standard oligonucleotides is by using the nicking enzyme strategy that we also demonstrate as a way to produce ssDNA. Although in this case, it limits the design of constant regions used for PCR primers according to the use of chosen nicking enzymes. It may also cause the loss of ∼5% of sequences from random libraries when they fortuitously harbor the cleavage site. Also, as for RPIdAs and HEGL primers, this method is based on DNA strands of different sizes, requiring denaturing PAGE purification and thus limiting the approach to lengths that permit such separation (it would not allow separation of strands from a PCR amplicon of a kilobase for instance). On the other hand, in principle if a fixed sequence incorporating a nicking enzyme site recognition is used in the “middle region” of a longer sequence (e.g., 1 kb), this approach could be used to specifically cut in half the reverse strand and purify the forward strand following gel separation. Finally, even if purification by denaturing PAGE will clearly separate digested DNA strands from full length DNA, as seen in Figure 7B, the native gel at 37°C (Figure 7C) suggests that single strand DNA can readily be obtained by simply heating.

With all the potential applications of aptamers, and more generally ssDNA, including in synthetic biology, more options to efficiently produce ssDNA during the SELEX procedure are desirable. Moreover, herein we also describe some peculiarities of random library amplification, which may help provide a better basis to further improve the process in the future.

## Data Availability Statement

All datasets generated and analyzed for this study are included in the article/Supplementary Material.

## Author Contributions

AN and JP conceived and designed the study and performed the overall editing of the manuscript. AN and NS performed the experiments of ssDNA amplification using non-extendable primer or primer with internally inverted nucleotide. VA-S, AF, and EY performed experiments of ssDNA amplification using nicking endonucleases. AN, NS, JP, VA-S, AF, and EY collected the experimental data and performed data analysis. AN, NS, and JP wrote the manuscript. AN, JP, and MB oversaw the project.

## Conflict of Interest

The authors declare that the research was conducted in the absence of any commercial or financial relationships that could be construed as a potential conflict of interest.

## Supplementary Material

TABLE S1 List of oligonucleotides used in this study. ddC: dideoxycytidine; invdA: inverted (3′->5′) adenine; N: degenerated position (any nucleotide).
